# Alteration of payload in extracellular vesicles by crosstalk with mesenchymal stem cells from different origin

**DOI:** 10.1186/s12951-021-00890-9

**Published:** 2021-05-20

**Authors:** Dong Jun Park, Jeong-Eun Park, Tae Hoon Kong, Young Joon Seo

**Affiliations:** 1 Department of Otorhinolaryngology, Yonsei University Wonju College of Medicine, 20 Ilsan- ro, Wonju, Gangwon-do 26426 South Korea; 2 Research Institute of Hearing Enhancement, Wonju, Gangwon-do 26426 South Korea; 3 Department of Surgery, University of California San Diego, 212 Dickinson Street, MC 8236, San Diego, CA 92103 USA; 4 School of Pharmacy and Biomedical Sciences, Curtin University, Bentley, WA 6102 Australia

**Keywords:** Extracellular vesicles, Mesenchymal stem cell, Antiinflammation, Amyloid- A4 precursor protein-binding family A member 2, miR-638

## Abstract

**Background:**

The application of extracellular vesicles (EVs) derived from mesenchymal stem cells (MSCs) requires customized materials to target disease or cell damage. We hypothesized that EVs exert different inflammatory effects on one recipient cell, although stem cells of different origins in humans have similar payloads.

**Results:**

Here, the payload of EVs released by crosstalk between MSCs and human middle ear epithelial cells (HMEECs) extracted from adipose tissue, bone marrow and tonsils significantly increased the level of anti-inflammatory factors. EVs derived from the co-culture medium decreased *TNF-, COX-2, IL-1*, and *IL-6* levels to approximately zero within 3h in HMEECs. Expression of miR-638 and amyloid- A4 precursor protein-binding family A member 2 was analyzed using microarrays and gene ontology analysis, respectively.

**Conclusions:**

In conclusion, stem cells of different origins have different payloads through crosstalk with recipient-specific cells. Inducing specific factors in EVs by co-culture with MSCs could be valuable in regenerative medicine.

**Graphical abstract:**

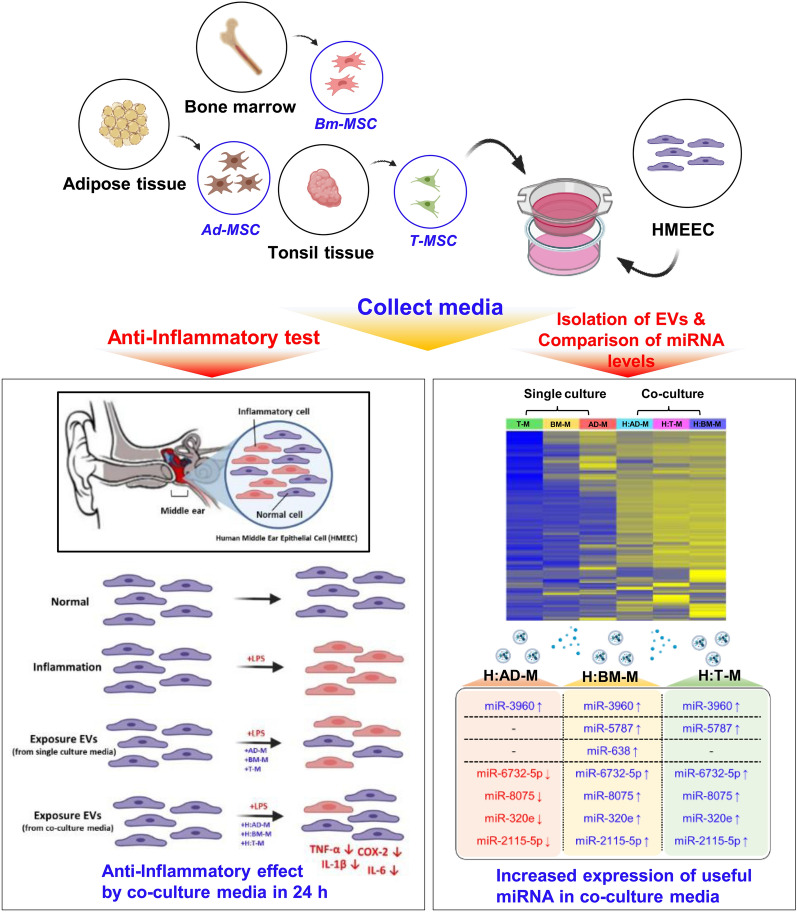

## Background

Otitis media (OM) is one of the most common diseases affecting children [1]. Although acute OM may resolve spontaneously, oral antibiotics and corticosteroids are ineffective in the long-term treatment of recurrent and chronic OM. Bacterial resistance to antibiotics has become an emerging problem in many parts of the world. Researchers have focused on understanding the pathophysiology of OM as a replacement for antibiotic treatment, including the role of inflammatory mediators and ion homeostasis molecules [2]. Restoration of fluid homeostasis function in the middle ear epithelium could be a target for future treatment, similar to the role of the upper respiratory ciliated mucosa. The middle ear mucosa is a pseudostratified, ciliated columnar epithelium through the eustachian tube that continues to the respiratory tract. Stem cell-derived exosomes control inflammatory signaling within the airway through intercellular communication, as demonstrated by the transfer of suppressor of cytokine signaling 1 from macrophage-derived exosomes to alveolar epithelial cells [3]. Human mesenchymal stem cell (hMSC)-derived extracellular vesicles (EVs) possess most of the anti-inflammatory and neuroprotective activities of MSCs in the respiratory epithelium [4, 5].

Therapeutics based on stem cell technology, including stem cell-derived EVs, have emerged in recent years and can treat what were otherwise considered incurable diseases in the field of cancer or cell regeneration [5]. Studies have also been proposed to find biomarkers in EVs for specific cancers [6]. Mesenchymal stem cells (MSCs) have been found to have different sources, such as the bone marrow, fat, urine, and tonsil; although they have the same phenotype, they are reported to have different internal payloads with various efficacies of treatments [5, 7]. It has been found that tumor-derived EVs play a key role in crosstalk between malignant and transformed cells and the immune system and that cancer cells suppress immune surveillance [8]. In addition, stem cell-derived EVs are involved in crosstalk with surrounding cells during development and release their molecules to them [9, 10], and help the survival of surrounding cells through secretion via EVs of inflammatory, differentiation, and proliferation factors [4, 5].

EVs are a heterogeneous group of secreted membranous vesicles, including microvesicles, ectosomes, and exosomes [11]. They have become valuable biomarkers in liquid biopsies [12], and existing research has focused on their characterization in different cancer types [9]. When stem cells undergo autophagy through external stimulation, exocytosis is stimulated when intracellular multivesicular bodies (MVBs) increase, resulting in a large number of extracellular vesicles [13]. Furthermore, EVs are known to be released by a variety of human cells and are important mediators in the coordination of the immune response to maintain host homeostasis [5, 14]. Upon different stimulation, EVs carry different combinations of nucleic acids, proteins, and lipids to other cells [15] and have been reported to be associated with certain pathological conditions, particularly microbial infections and cancer [16]. It has also been reported that the amount of EVs released following external stimulation increases with time [13]. The ability to block the transfer of tumor-derived EVs containing oncogenic messages, such as EGFR, into recipient cells is a potential antitumor strategy. A study showed that incubation of EVs from a cell line with heparin blocked their transfer into recipient cells [17].

In this study, we evaluated the anti-inflammatory effect of EVs derived from hMSCs extracted from the adipose tissue, bone marrow, and tonsils. MSCs derived from these sources contain several factors related to cell proliferation and inflammation-reducing substances [18,20], but miRNA or cytokine expression and efficacy by crosstalk with new recipient cells may differ [14, 21]. Various studies have reported the relief of inflammation in skin tissues and infected organs in animal models [22]. The current focus of anti-inflammatory studies using MSC-derived EVs is to increase the payload of useful factors in EVs through discovery of factors through wound healing and mechanism research [23, 24]; there are reports that a calcium-dependent mechanism increases the release of EVs from the cells [25]. However, the important aspect of MSC research is to observe the change in payload inside the MSC and understands its role in disease [21]. In this study, we profiled the factors related to anti-inflammatory activity in EVs derived from MSCs extracted from different origins, when co-cultured with human middle ear epithelial cells (HMEECs), which can cause OM [26]. Interestingly, the expression level of molecule payload and anti-inflammatory efficiency were different in EVs from MSCs derived from adipose tissue, bone marrow, and tonsils co-cultured with HMEECs. Therefore, we propose a technology to discover new miRNA candidates by profiling it to prove inflammation relief according to the change of EV payload by crosstalk between MSCs and target cells is altered. In addition, the technique, involving the co-culture condition, offers a new direction for MSC-derived EV research to cure diseases or cancer; studies on the mechanism underlying the crosstalk between donor and recipient cells are also needed.

## Materials and methods

### Extraction of mesenchymal stem cells (MSCs) derived from adipose tissue, bone marrow, and tonsils

The human adipose tissue, bone marrow, and tonsils were obtained from the iliac crest of patients who received transplantation treatment at the Wonju Severance Christian Hospital after obtaining their written consent (IRB number: CR320104). The extraction of adipose tissue-derived MSCs was performed as described by Cho et al. [18]. Briefly, the tissue samples were washed twice with phosphate-buffered saline (PBS, D8537, Sigma-Aldrich, MA, USA) to remove blood cells and incubated with 200 IU/mL collagenase (Thermo Fisher Scientific, Carlsbad, CA, USA) for 45min at 37C. After incubation, the samples were centrifuged at 300*g* for 7min, the supernatant was discarded, and the remaining pellets were resuspended in MEM-alpha (Hyclone, Logan, UT, USA) with 10% fetal bovine serum (FBS) (Gibco, Grand Island, NY, USA) and antibiotics (anti-anti, Thermo Fisher Scientific, Carlsbad, CA, USA). The cells were transferred to a culture plate and incubated in a humidified atmosphere of 95% air and 5% CO_2_ at 37C.

To extract MSCs derived from the bone marrow, aspirates were collected into Vacutainers K2 EDTA (BD Biosciences, San Jose, CA, USA). The mononuclear cells were diluted 1:5 with PBS and separated by density gradient centrifugation at 435*g* for 20min at room temperature (RT, 25C) using a Ficoll Hypaque (GE17-1440-02, Gibco, Grand Island, NY, USA) solution. The cell fractions were collected and cultured using MEM-alpha with 10% FBS and antibiotics at a seeding density of 510^3^ cells per cm^2^. The plate was maintained at 37C in a humidified atmosphere containing 5% CO_2_. To exchange the medium, the plate was washed with PBS to remove the non-adherent cells, and the medium was replaced. Upon reaching 70% confluence, the cells were passaged to 110^6^ cells/plate.

Tonsil-derived MSCs were extracted as described by Bacic et al. [27]. The tonsil tissue was gently washed with ethanol and cut with surgical scissors on a plate. After washing with PBS, the tissue mixture was added to Falcon tubes and incubated with PBS, 200 IU/mL collagenase, and 10g/mL DNase (EN0525, Thermo Fisher Scientific, Carlsbad, CA, USA) at 37C in a water bath for 1h. After filtering the suspension using a cell strainer, the monocytes were isolated from the supernatant using Ficoll Hypaque density gradient centrifugation. The cell fractions were collected and cultured using MEM-alpha with 10% FBS and antibiotics at a seeding density of 510^3^ cells per cm^2^.

### Flow cytometric analysis

Flow cytometry was used to assess the immune profile of MSCs using the standard for MSCs, as described by the International Society for Cellular Therapy (ISCT) [28]. The cell surface markers were analyzed using a human MSC (hMSC) analysis kit (562245, BD Biosciences, San Jose, CA, USA). According to the manufacturers instructions, the hMSC-positive cocktail (CD90 FITC, CD105 PerCP-Cy5.5, and CD73 APC) and PE hMSC negative cocktail (CD34, CD11b, CD19, CD45, and HLA-DR) were used as the positive and negative controls, respectively. As stated in this recommendation in the manufacturers instructions, MSCs should be positive for CD73, CD90 and CD105, but negative for CD34, CD45, CD11b or CD14, CD19 or CD79 and HLA-DR. Since MSCs are known to express numerous cell surface markers such as CD44, CD29, CD200, CD166, CD146, and CD271, we used CD44 as a representative in this study. The hMSC Positive Isotype Control Cocktail (mIgG1 FITC, mIgG1 PerCP-Cy5.5, and mIgG1 APC) and PE hMSC Negative Isotype Control Cocktail in the kit (mIgG1 PE and mIgG2a PE) were also used as an isotype control for the analysis. The samples were analyzed through flow cytometry using a FACS Aria3 flow cytometer (Becton Dickinson, San Jose, CA, USA). The data were analyzed using the FACS Diva software.

### Culture of human middle ear epithelial cells

HMEECs were provided by Dr. David J. Lim (House Ear Institutes, Los Angeles, CA, USA). The cells were immortalized with the E6/E7 genes of the human papillomavirus [29] and cultured in Dulbeccos modified Eagles medium (Lonza, MD, USA) and bronchial epithelial basal medium (Lonza, 1:1). The medium was changed every 3 days after washing with PBS. The cells were then humidified at 37C with 5% CO_2_.

### Isolation of extracellular vesicles

To obtain EVs, initial extraction was performed according to the MISEV guidelines as far as possible [30]. The cells were cultured in a serum-free environment for inducing EV expression, and analyzed EVs from the media by western blot and NTA. After centrifuging the cells and debris, the medium was collected and centrifuged at 2000*g* for 30min, and the total exosome isolation reagent (4478359, Invitrogen, Grand Island, USA) was used according to the manufacturers instructions. To purify EVs from the fractions, we centrifuged the fraction at 10,000*g* for 1h at 4C on the next day and added 200 L of PBS to the sunk pellets to dissolve them. The samples were centrifuged at 22,000*g* for 2h at 4C using a table microcentrifuge (5424R, Eppendorf, Germany) to obtain high-purity EVs.

### Transwell study

Co-culture conditions employed in this study were reported by Lu et al. [31]. One million MSCs were seeded into a transwell insert with a 0.4-m pore size (3401, Corning, NY, USA) consisting of a polycarbonate membrane. MSCs derived from adipose tissue, bone marrow, and tonsil were co-cultured with HMEECs in the inner well. In addition, HMEECs were seeded into the outer well. They were then carefully transferred and cultured in a 37C incubator with 5% CO_2_ in a humidified atmosphere prior to the appropriate LPS treatment or isolation of EVs from the media.

### Cell viability assay

Cell viability was measured using a cell counting kit according to the manufacturers instructions (CCK-300, Seoul, Korea). The cells were seeded at 110^3^ cells per well in a 96-well plate. After exposure to 1, 5, 10, and 100g/mL LPS for different periods of time, CCK-8 solution was added to each well and the plates were incubated for 4h at 37C. Cell viability was also measured after HMEECs were co-cultured with MSCs for 24h. The plate was mixed thoroughly using a shaker, and the optical density was measured at 450 nm using a microplate reader (Epoch, BioTek, VT, USA).

### Real-time PCR

To evaluate the expression of the inflammatory markers, total RNA was isolated from HMEECs after treatment with extracellular vesicles using TRIzol reagent (Invitrogen, Carlsbad, CA, USA). RNA was reverse-transcribed using a ReverTra Ace qPCR RT Master Mix (Toyobo Bio-Technology, Osaka, Japan). The following primers were used for sequencing: *TNF-*, forward: 5-GA GGC CAA GCC CTG GTA TG-3 and reverse: 5-CG GGC CGA TTG ATC TCA GC-3; *COX-2*, forward: 5-TT GCT GGC AGG GTT GCT GGT-3 and reverse: 5-TC TGC CTG CTC TGG TCA ATG G-3; *interleukin 1 (IL-1)*, forward: 5-TC CAG GGA CAG GAT ATG GAG-3 and reverse: 5-CC AAG GCC ACA GGT ATT TTG-3; *interleukin 6 (IL-6)*, forward: 5-AA AGA GGC ACT GGC AGA AAA-3 and reverse: 5-AG CTC TGG CTT GTT CCT CAC-3; and *GAPDH*, forward: 5-TC GCC CCA CTT GAT TTT GG-3 and reverse: 5-GC AAA TTC CAT GGC ACC GT-3. The thermal cycling conditions comprised an initial denaturation at 95C for 15min, followed by 33 cycles of 94C for 30s, 50C for 30s, and 72C for 60s. Real-time PCR was performed using the QuantStudio 6 Flex Real-Time PCR System (Applied Biosystems, Foster City, CA, USA).

### Confocal microscopy

Reactive oxygen species (ROS) were detected using 1 M 2,7-dichlorodihydrofluorescein diacetate (DCFDA) in HMEECs. The cells were then incubated in a humidified 5% CO_2_ incubator for 30min at 37C after treatment with DCFDA. In contrast, CD63, an EV marker, was observed when the MSCs were incubated in 5% normal goat serum for 1h at RT to prevent non-specific labeling. Anti-CD63 (1:200, ab 118,307, Abcam, MA, USA) was used as the primary antibody for 1h at 4C. The samples were washed with PBS three times for 5min each time, followed by incubation with a secondary antibody, goat anti-rabbit IgG H&L (Alexa Fluor 488; 1:1000, ab150077, Abcam, MA, USA), for 1h at RT. After washing the samples three times for 5min with PBS, they were immobilized with a mounting solution containing DAPI (4,6-diamidino-2-phenylindole). All the samples were observed by confocal microscopy (Carl Zeiss Microscopy GmbH, Jena, Germany), and the images were analyzed using ZEN lite ver. 2.3.

### Nanoparticle tracking analysis (NTA)

Extracellular vesicles were quantified based on the MISEV guidelines [30]. The particle concentration, size, and distribution of the isolated MSC-derived EVs were analyzed using a NanoSight NS300 (Malvern Instruments Ltd, Malvern, UK). Typically, 1 mL of a 1:100 diluted MSC-derived EV was prepared for particle visualization and recording of light scattering. Three videos, each of 60s recordings, were analyzed and plotted to show the EV concentration, size, and distribution.

### Transmission electronic microscopy (TEM)

MSCs were fixed in 2.5% glutaraldehyde for 2h at 4C, and the samples were solidified in 2% agar. After washing with 0.1M cacodylate buffer, the samples were post-fixed in 1% osmium tetroxide (OsO_4_). The dehydration steps were performed with 50100% ethanol, and the samples were then embedded in Epon resin. The samples were baked overnight in an oven at 65C, sectioned in an ultramicrotome, and examined by TEM using a field electron emission unit (JEM-1200EX-II, JEOL).

### Western blotting

EVs derived from MSCs were isolated through centrifugation, and the amount of total protein was quantified using the Bradford assay. Protein samples were separated using 10% SDS-PAGE with a mini gel apparatus (Bio-Rad, Hercules, CA, USA) and transferred onto PVDF membranes (T831.1, Merck Millipore, MA, USA). Each membrane was blocked with 3% skim milk in Tris-buffered saline containing 0.05% Tween 20. The primary antibodies anti-CD9 (1:1000, ab92726, Abcam, MA, USA), CD63 (1:1000, ab118307, Abcam, MA, USA), and anti-CD81 (1:1000, ab109201, Abcam, MA, USA) were used as primary antibodies and were incubated overnight at 4C. Mouse anti-rabbit IgG-HRP (sc-2357, Santa Cruz, CA, USA) was used as a secondary antibody for 1h. The bands were visualized using enhanced chemiluminescence according to the manufacturers instructions (Immobilon Crescendo Western HRP substrate, Millipore, Darmstadt, Germany). The band intensities were quantified using the ChemiDoc XRS+System (Bio-Rad). All the samples were developed within 10min to obtain a band.

### miRNA analysis

RNA samples prepared from EVs were isolated from the single and co-culture media. The RNA from the EVs was labeled AD-M, BM-M, and T-M derived from single cultured media, such as adipose tissue (AD)-derived MSCs, bone marrow (BM)-derived MSCs, and tonsil (T)-derived MSCs. The RNA from the EVs was labeled as H:AD-M, H:BM-M, and H:T-M derived from co-culture media as AD-MSCs, BM-MSCs, and T-MSCs with HMEECs. The concentration of the sample was confirmed through quality control analysis and was confirmed to be between 0.13 and 0.28 ng/L. To check the purity and quantity of RNA, a NanoDrop spectrophotometer was used to measure the absorbance at 260 and 280 nm. All raw data were extracted automatically using the Affymetrix data extraction protocol using the Affymetrix GeneChip Command Console Software (AGCC). The CEL files were imported, miRNA levels were normalized using the RMA algorithm, the detection above background p-values was calculated for all data, and the results were exported using the Affymetrix Power Tools (APT) software. Array data were filtered using species-specific annotated probes. Comparative analysis between the test and control samples was performed using fold change. All statistical testing and visualization of differentially expressed genes were conducted using the R statistical language 3.3.3 (https://www.r-project.org/). All the predicted mRNAs from the miRNAs in the EVs were analyzed using the NCBI database, ExoCarta (http://www.exocarta.org/), and Vesiclepedia (http://microvesicles.org/).

### Statistical analysis

Statistical analysis was performed using the SPSS statistical package (version 21.0; SPSS Inc., USA). All graphs were plotted using GraphPad PRISM (version 5.0; GraphPad Inc., La Jolla, CA, USA). The descriptive results of continuous variables are expressed as meanstandard deviation (SD) for normally distributed variables. The means were compared using a two-way analysis of variance. The level of statistical significance was set at p<0.05.

## Results

### Validation of mesenchymal stem cells derived from adipose tissue, bone marrow, and tonsil using FACS

**Fig. 1:**
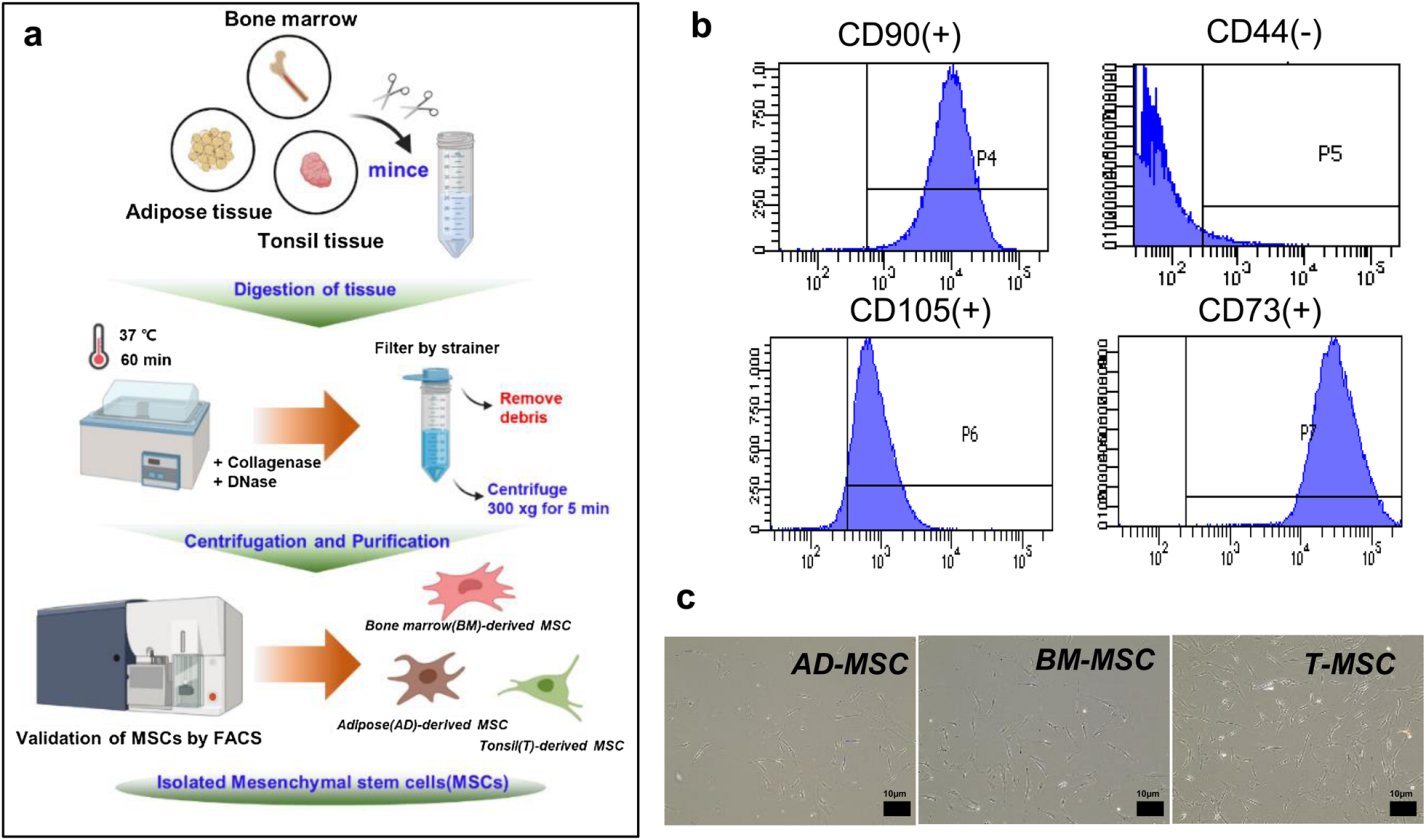
Extracted and validated MSCs from the adipose tissue, bone marrow, and tonsil tissue in humans

To confirm the effects of MSCs in various locations in the human body, we obtained MSCs from three different human tissues. Adipose tissue-, bone marrow-, and tonsil-derived MSCs have been reported to have cell proliferation and inflammation alleviation efficacy in various studies [4]. We established conditions for purely separating primary cultured MSCs from fresh tissues, and all the samples were validated with specific markers using FACS (Fig.1a). All the tissues were collected from human donors at our institute. As shown in Fig.1b, the expression of CD markers in MSCs was demonstrated as CD90, CD105, and CD73, and CD44 in FACS. The MSCs derived from adipose tissue, bone marrow, and tonsils showed similar morphology using microscopy (Fig.1c).They were labeled AD-MSC, BM-MSC, and T-MSC since they are MSCs derived from the adipose tissue, bone marrow, and tonsil, respectively (a) The process of extracting and validating MSCs from three tissues, (b) The validation result of MSCs by FACS. MSCs were positive for CD90, CD105, and CD73 and negative for CD44, (c) Microscopic image of AD-MSC, BM-MSC, and T-MSC (bar =10m).

### Increased levels of inflammatory markers by LPS on human middle ear epithelial cells

**Fig. 2:**
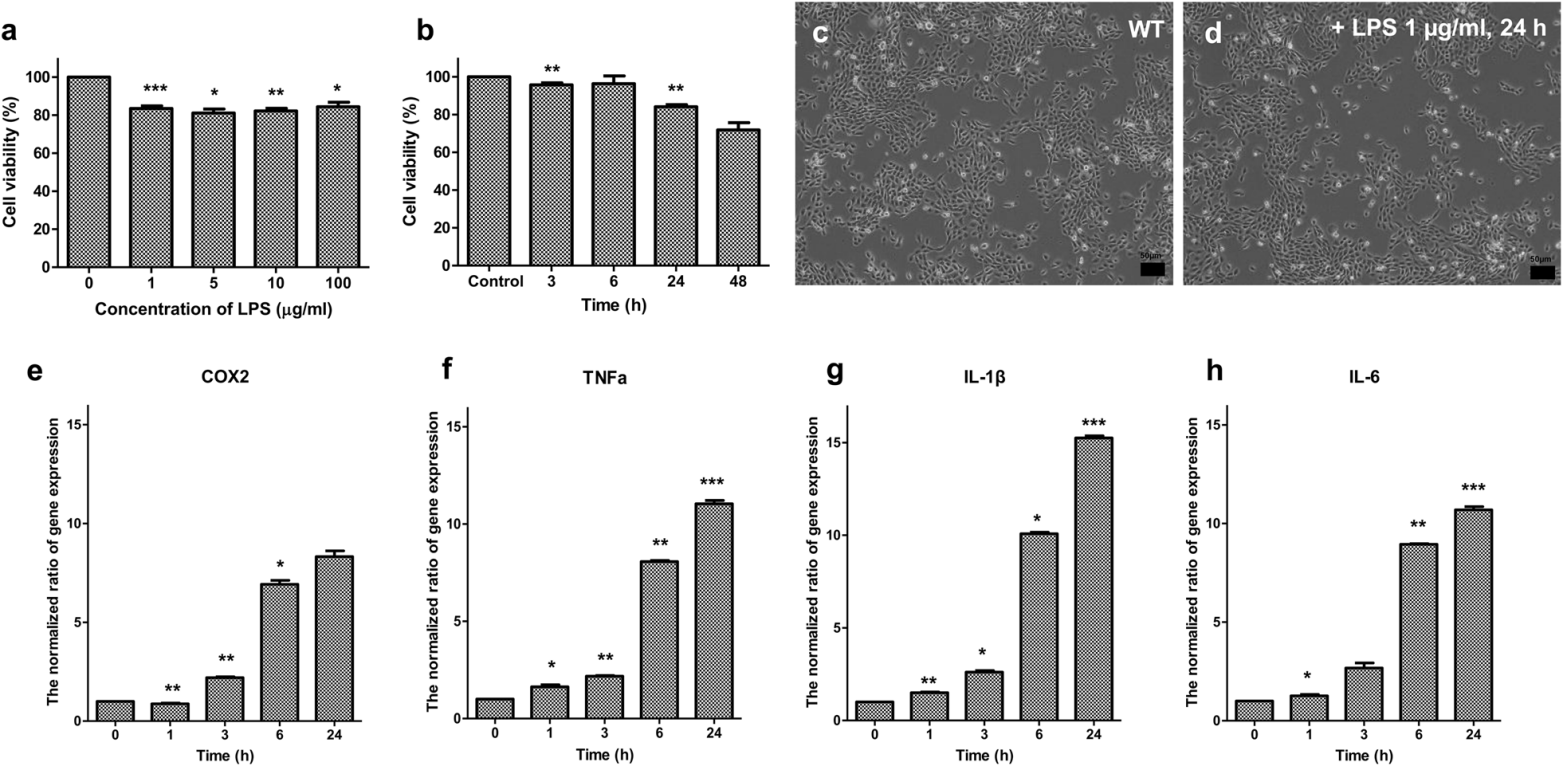
Cell viability and expression of *TNF-, COX-2, IL-1*, and *IL-6* in HMEECs upon LPS treatment. **a** The viability of HMEECs after treatment with 1, 5, 10, and 100g/mL LPS for 24h, **b** The viability of HMEECs treated with 1g/mL LPS for 1, 3, 6, 24, and 48h, **c** Image of wild-type HMEECs; **d** Image of HMEECs after treatment with 1g/mL LPS for 24h, **e** *COX-2* expression level in HMEECs after treatment with 1g/mL LPS for 24h, **f** *TNF-* expression level in HMEECs after treatment with 1g/mL LPS for 24h, **g** *IL-1* expression level in HMEECs after treatment with 1g/mL LPS for 24h, **h** *IL-6* expression level in HMEECs after treatment with 1g/mL LPS for 24h (p-value: ***<0.001, **<0.01, *<0.05)

Anti-inflammation research using MSCs has been reported in various fields [4]. In this study, we aimed to compare the anti-inflammatory effect of MSCs derived from three different tissues; therefore, we decided to use an in vitro model that can be used for a clinical study related to inflammation. HMEECs are used in OM research because they are easy to access as an initial model to prove the effect between anti-inflammatory reactions with stem cells [26]. We selected the effective concentration (FC20) that showed 81% from 1g/mL viability after 24h (Fig.2a) and 83% after 24h with 1g/mL LPS (Fig.2b) because HMEECs must be maintained to extract cell-derived RNA to measure inflammatory factors. Cell imaging via microscopy showed that dead cells were observed by LPS (Fig.2c and d). To confirm the expression of inflammatory markers, we designed four primers (*COX-2, TNF-, IL-1, and IL-6*) for real-time PCR and found that the expression levels of these markers were increased by 1g/mL LPS for 24h. The expression level increased rapidly after 6h and remained constant after 24h (Fig.2eh).

### Evaluated cell viability and ROS reduction in the co-culture condition between HMEEC and MSC derived from adipose tissue, bone marrow, and tonsils

**Fig. 3:**
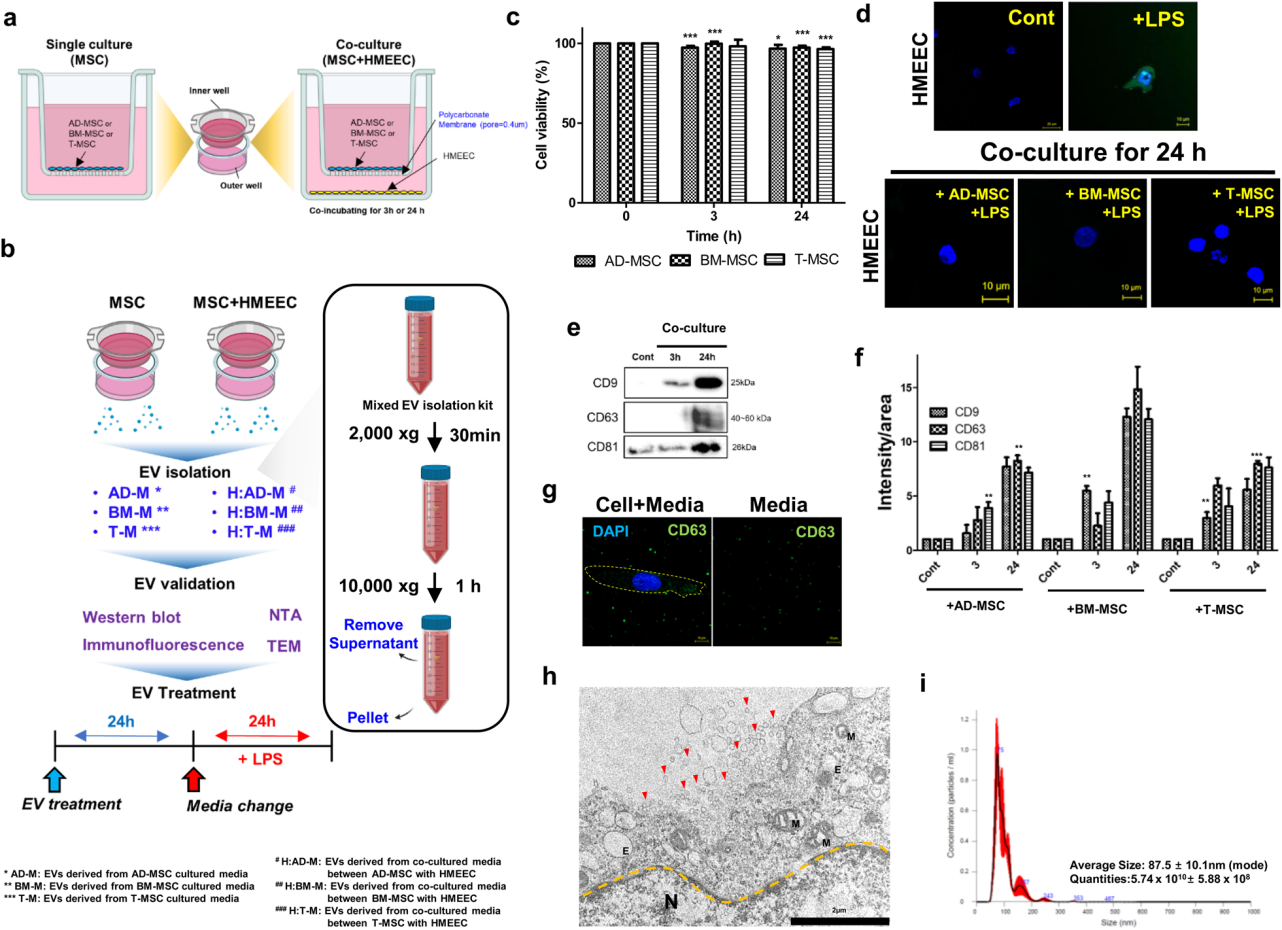
Schematic diagram of co-culture conditions and extraction and evaluation of MSC-derived EVs. **a** Scheme of culture condition, **b** Flow chart for EV isolation and treatment of HMEECs, **c** The viability of HMEECs when co-cultured with AD-MSC, BM-MSC, and T-MSC for 3 and 24h (p-value:***<0.001, **<0.005, *<0.05), **d** Evaluation of ROS reduction in HMEECs treated with LPS after co-culture with AD-MSC, BM-MSC, and T-MSC for 24h, **e** The expression of CD9, CD63, and CD81 protein bands in the co-culture media, **f** The normalized intensity of CD9, CD63, and CD81 with the area in the co-culture media (p-value: ***<0.001, **<0.005, *<0.05), **g** The expression of CD63 as an EV marker in the MSC culture media (yellow circle represents the cellular membrane), **h** The image of EVs (red arrow) released from the MSC, **i** The size and concentration analysis in EVs by NTA

Since it is difficult to directly confirm the anti-inflammatory effect of donor cells using MSCs, we tried to prove the anti-inflammatory effect using EVs generated from the co-culture medium. EVs have been reported to occur even in MSC single cultures [32], but the effect of EVs in the co-culture media has not been reported. We hypothesized that since EVs play a role in cell-cell communication, internal substances (miRNAs, proteins, cytokine etc.) change because of the crosstalk between cells [21, 33]. Therefore, adipose-derived MSCs (AD-MSCs), bone marrow-derived MSCs (BM-MSCs), and tonsil-derived MSCs (T-MSCs) were cultured alone. The EVs in the single culture media were labeled as AD-M, BM-M, and T-M. In addition, AD-, BM-, and T-MSCs were co-cultured with HMEECs in a transwell plate to obtain a co-culture medium. The EVs in the co-culture media between MSCs and HMEECs were labeled H:AD-M, H:BM-M, and H:T-M (Fig.3a). All the EVs were purified using an exosome isolation kit with high centrifugation and were validated in various ways. The efficacy of the EVs was compared after inducing inflammation with LPS in HMEECs pre-treated with EVs (Fig.3b).

Cell viability was evaluated, as shown in Fig.3c. When HMEECs were co-cultured with AD-MSCs, BM-MSCs, and T-MSCs, the cell viability was maintained at over 98% for 3 and 24h. In other words, it was found that HMEECs and MSCs do not cause toxicity to each other through crosstalk. Interestingly, it was also observed that the ROS levels were reduced by the three MSCs after ROS production was stimulated by LPS in HMEECs (Fig.3d). This suggests that they can be reduced by substances generated during co-culture with MSCs; therefore, it was hypothesized that the ROS-reducing substances in the co-culture medium were EVs. In addition, the amount of protein was evaluated in EVs through specific EV markers, such as CD9, CD63, and CD81 in co-culture media, and it was observed that the protein expression level gradually increased at 3 and 24h (Fig.3e). When co-cultured with AD-MSCs, BM-MSCs, and T-MSCs, the ratio of the amount of protein showed a similar tendency even when the amount of protein was quantified by area (Fig.3f). The fluorescence images of EVs were analyzed in MSC culture media using the EV marker CD63 through immunofluorescence (IF) (Fig.3g). Green fluorescence intensity was detected in the media, except cells, and the phenome of EVs released from the MSC surface was observed through TEM (Fig.3h). In addition, the EVs dissolved in the media were measured by NTA; the EV size was 87.510.1 nm, and the concentration of EV was 5.7410^10^ 5.8810^8^ particles/mL (Fig.3i). Therefore, we observed that the three MSCs and HMEECs could be co-cultured without toxicity by crosstalk and it can be predicted that a substance that reduces ROS is contained in the EV.

### Decreased TNF-, COX-2, IL-1, and IL-6 in co-culture media

**Fig. 4:**
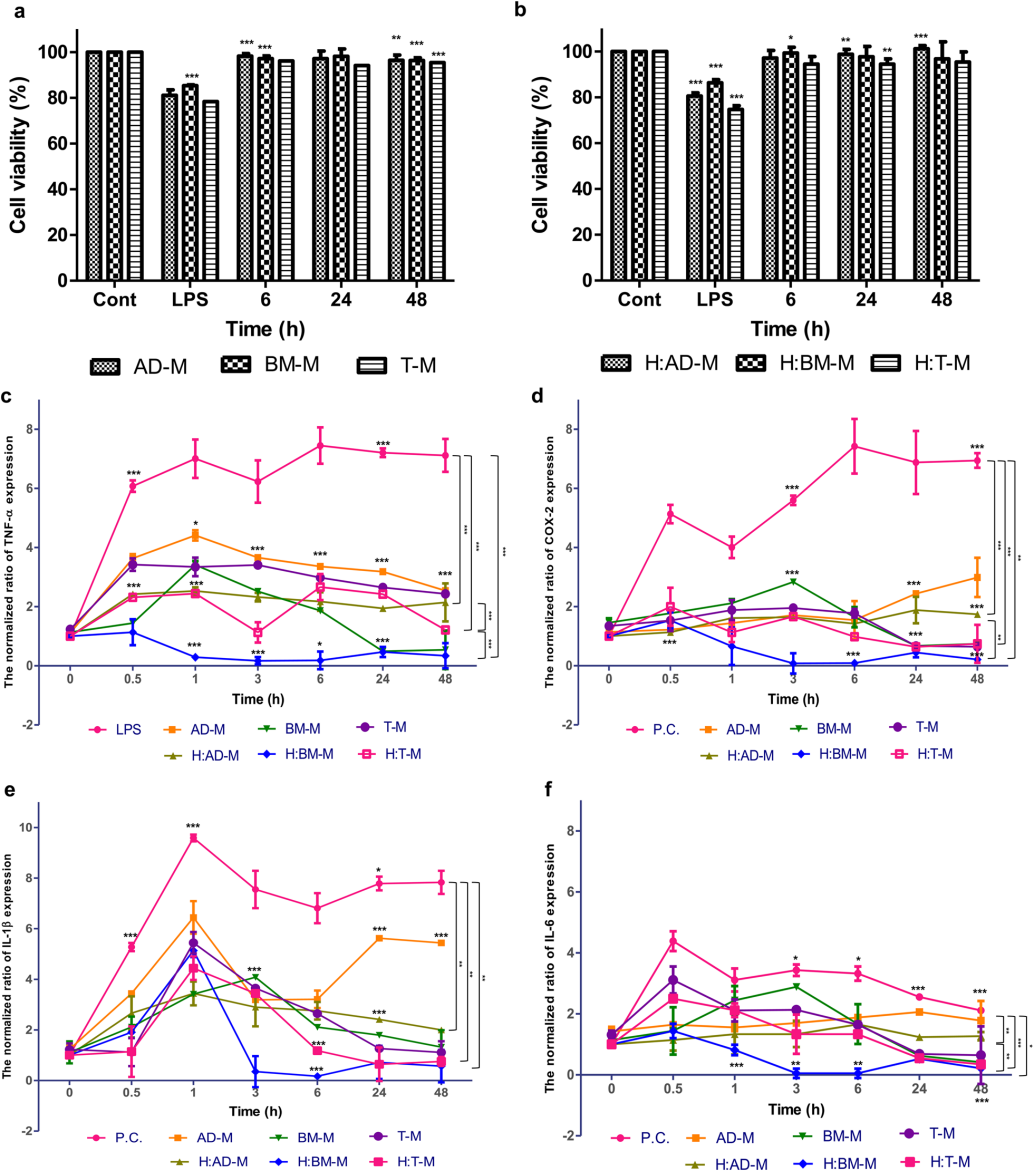
Evaluation of inflammatory markers (*TNF-, COX-2, IL-1*, and *IL-6*) using EVs derived from the three different co-culture media. **a** The viability of HMEECs at 6, 24, and 48h after treatment with EVs (AD-M, BM-M, and T-M) derived from single culture media, **b** The viability of HMEECs at 6, 24, and 48h after treatment with EVs (H:AD-M, H:BM-M, and H:T-M) derived from co-culture media, **c** *TNF-* expression level in HMEECs after treatment with six EVs with 1g/mL LPS until 48h (p-value: ***<0.0001), **d** *COX-2* expression level in HMEECs after treatment with six EVs with 1g/mL LPS until 48h (p-value: ***<0.0001, **<0.005), **e** *IL-1* expression level in HMEECs after treatment with six EVs with 1g/mL LPS until 48h (p-value: **<0.005), **f** *IL-6* expression level in HMEECs after treatment with six EVs with 1g/mL LPS until 48h (p-value: ***<0.001, **<0.005, *<0.05)

ROS levels were reduced by co-culture with the three MSCs, but we isolated EVs from the media. When the HMEECs were co-cultured with the three MSCs and exposed to LPS, LPS stimulated both the MSCs and HMEECs. To evaluate the efficacy of the purified EVs, they should be isolated from the media and compared with single culture media. The viability of HMEECs was evaluated using EVs isolated from single culture media (AD-M, BM-M, and T-M) and EVs extracted from co-culture media (H:AD-M, H:BM-M, and H:T-M). When LPS was treated at 1mg/mL for 24h as EC20, the cell viability was approximately 81%. In contrast, the viability of HMEECs treated with AD-M, BM-M, and T-M for 648h was 98% (Fig.4a). It also showed that the viability of HMEECs was more than 97% when treated with H:AD-M, H:BM-M, and H:T-M for 648h (Fig.4b). Since the stability of the extracted EVs was confirmed, we compared the effect of inflammatory factors in HMEECs that induced inflammation by EC20 concentration of LPS.

To evaluate the anti-inflammatory effect of EVs isolated from single and co-culture media, the RNA expression level was confirmed through real-time PCR. To confirm the anti-inflammatory effect of EVs extracted from a single culture medium and co-culture medium, the expression of RNA inflammatory factor generation was observed using real-time PCR. After treating with EVs, the HMEEC cultured media was changed and treated with LPS for 0.5, 1, 3, 6, 24, and 48h. EVs derived from single culture media, AD-M, BM-M, T-M, showed decreased levels of TNF- after 1h of exposure, which were 7.00.64 to 4.410.17 by AD-M, 3.410.13 by BM-M, and 3.340.31 by T-M (Fig.4c). In contrast, EVs derived from the co-culture medium H:AD-M, H:BM-M, and H:TM showed decreased levels of TNF- after 1h of exposure, which were 2.530.12 by H:AD-M, 0.290.04 by H: BM-M, and 2.440.04 by H:T-M (Fig.4c). Interestingly, in the case of EVs derived from co-culture media, the TNF- expression level did not increase within 1h by H:BM-M (Fig.4c). H:AD-M and H:T-M showed similar expression levels to AD-M and T-M. The expression level of COX-2 stimulated by LPS confirmed that BM-M and T-M showed decreased expression levels after 24h rather than AD-M.

It was also confirmed that the expression of COX-2 was reduced by co-culture medium-derived EVs. EVs derived from single culture media, AD-M, BM-M, and T-M, showed that the expression level of COX-2 decreased after 3h of exposure, which were 5.590.16, 1.710.03, 2.83002, and 1.950.01, respectively. In contrast, EVs derived from the co-culture medium H:AD-M, H:BM-M, and H:TM decreased the expression level of COX-2 after 3h. t was confirmed that the expression level of COX-2 also decreased significantly which showed 1.640.08 by H:AD-M, 0.080.34 by H: BM-M, and 1.660.01 by H:T-M after 1h (Fig.4d). In addition, it was confirmed that the IL-1 expression level decreased after 3h by BM-M and T-M, but not by AD-M. EVs derived from single culture media, AD-M, BM-M, and T-M, showed that the expression level of IL-1 decreased after 3h exposure, which were 7.550.73 to 3.180.02 by AD-M, 4.080.04 by BM-M, and 3.640.01 by T-M (Fig.4e). In contrast, EVs derived from the co-culture media H:AD-M, H:BM-M, and H:TM decreased the expression level of IL-1 after 3h, which were 2.890.75 by H:AD-M, 0.350.611 by H: BM-M, and 3.440.13 by H:T-M. As a result, by EVs derived from the co-culture medium, IL-1 decreased after 1h, and in particular, H:BM-M significantly reduced the expression of IL-1b after 3h (Fig.4e). The expression level of IL-6 was also reduced by EVs derived from MSC culture media after stimulation by LPS. EVs derived from single culture media, AD-M, BM-M, and T-M, showed that the expression level of IL-6 decreased after 3h exposure, which were 3.430.18 to 1.70.04 by AD-M, 2.880.04 by BM-M, and 2.130.02 by T-M (Fig.4f). In contrast, EVs derived from the co-culture media H:AD-M, H:BM-M, and H:TM decreased the expression level of IL-6 after 3h, which were 1.330.42 by H:AD-M, 0.0490.15 by H: BM-M, and 1.330.64 by H:T-M. Almost all expression levels decreased in the positive control, the AD-M, BM-M, and T-M groups showed no decrease in gene expression levels after 48h. However, IL-6 was significantly decreased after 30min by H:BM-M, and the expression level decreased after 3h (Fig.4f).

### Increased miRNA expression level in EVs by co-culture condition

The inflammatory factors were confirmed to significantly decrease in a short time in H:BM-M; therefore, we hypothesized that it would have been reduced by the factor possessed by EVs. In particular, in H:BM-M, it was hypothesized that the payload of a specific factor increases, and some studies have reported that some of the miRNA levels may vary depending on the co-culture conditions [9, 33, 34].

**Fig. 5:**
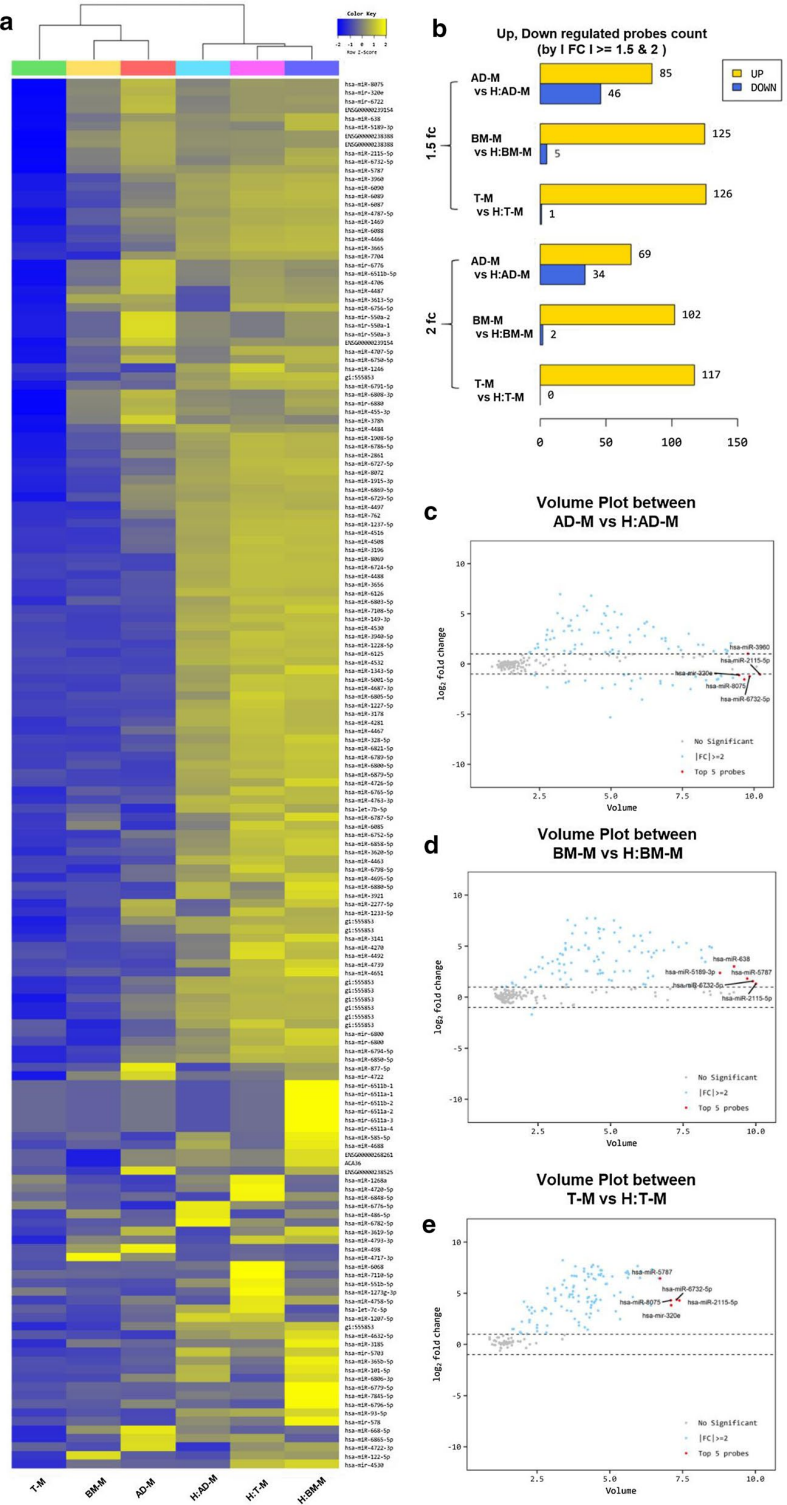
The data of miRNA analysis. **a** The heatmap of miRNA expression in EVs (AD-M, BM-M, T-M, H:AD-M, H:BM-M, and H:T-M), **b** The comparison of probe count in up- and downregulation between the six EVs, **c** The volume plot between AD-M and H:AD-M, **d** The volume plot between BM-M and H:BM-M, **e** The volume plot between T-M and H:T-M

As a result of miRNA analysis by EVs derived from cultured media in six different environments, we confirmed that a total of 161 miRNA expression levels changed (Fig.5a). Interestingly, as shown in the heatmap, it was confirmed that the miRNA level increased significantly under co-culture conditions. The miRNA expression level was compared by a fold change value of 1.5 and 2 times (Fig.5b). At 1.5 times, the expression of miRNA in AD-M and H:AD-M showed an increase of 85 miRNAs and a decrease of 46 miRNAs. The expression level of miRNA in BM-M and H:BM-M showed an increase of 125 miRNAs and a decrease of 5 miRNAs. The expression levels of miRNAs in T-M and H:T-M showed an increase of 126 miRNAs and a decrease of 1 miRNA. In addition, the expression level of miRNA was analyzed as a 2-fold change. There were 69 miRNAs and 34 downregulated miRNAs in the AD-M and H:AD-M groups. There were 102 miRNAs and two decreased miRNAs in BM-M and H:BM-M. There were 117 upregulated miRNAs in the T-M and H:T-M groups.

Furthermore, to analyze the miRNAs increased by co-culture, we selected the top five miRNAs with a large increase in expression level. Comparing IAD-M and H:AD-M, the highest miRNA expression was hsa-miR-3960, hsa-miR-2115-5p, hsa-miR-320e, hsa-miR-8075, and hsa-miR-6732-5p (Fig.5c). In addition, in the results of comparing BM-M and H:BM-M, the highest miRNA expression was hsa-miR-638, hsa-miR-5787, hsa-miR-5189-3p, hsa-miR-6732-5p, and hsa-miR-2115- It is 5p (Fig.5d). Finally, the results of comparing T-M and H:T-M, the highest miRNA expression were hsa-miR-2115-5p, hsa-miR-5787, hsa-miR-6732-5p, hsa-miR-8075, and hsa-miR-320e (Fig.5e). Interestingly, miRNAs that overlapped with each other were found, and a large amount of miRNA was observed to increase in H:BM-M.

**Fig. 6:**
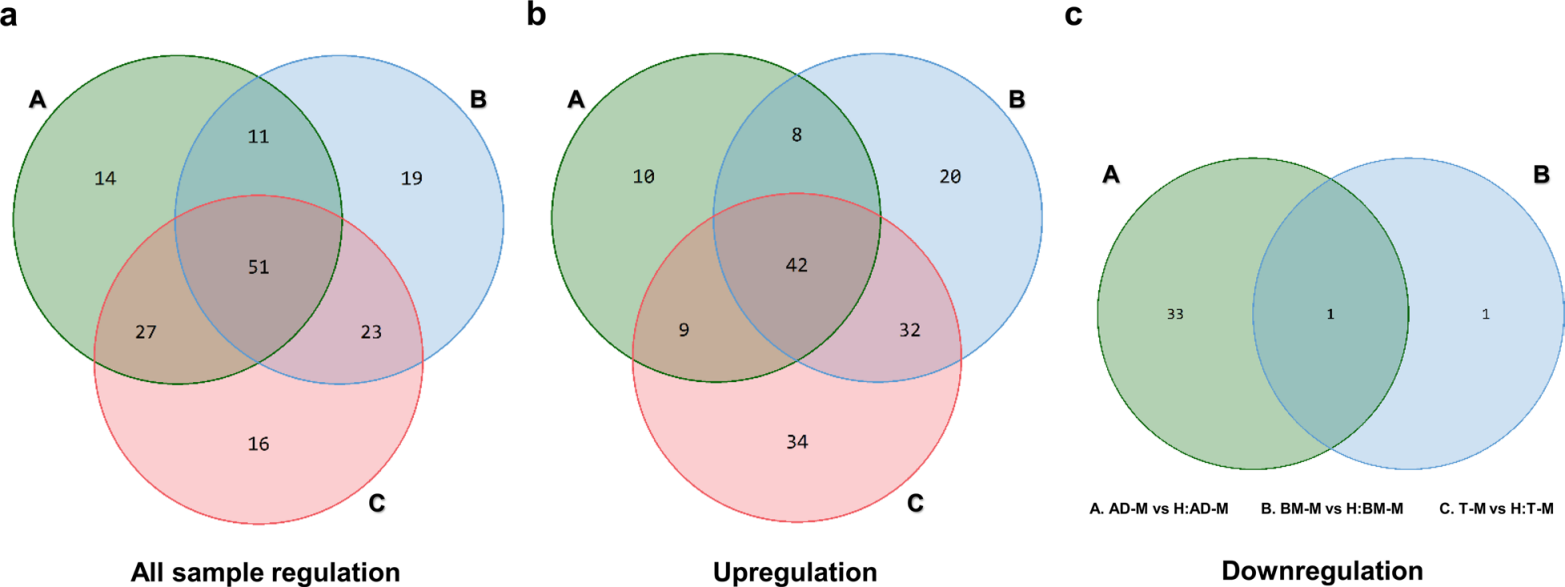
Venn diagram of logical miRNA relations in EVs (A, AD-M and H:AD-M; B, BM-M and H:BM-M; c, T-M and H:T-M). **a** Total regulation of all miRNA regulation for three groups, **b** Upregulation, **c** downregulation

To confirm the intersection of each EV sample from the three different MSCs, we performed a band diagram analysis and found that a total of 51 miRNAs were changed for overlapping factors under the three conditions (Fig.6a). In addition, the number of upregulated miRNAs was 42 under the three conditions (Fig.6b), and the decreased miRNA did not intersect in the TM vs. H:TM samples and one was found in AD-M vs. H:AD-M and BM-M vs. H:BM-M (Fig.6c).

### The amyloid-based binding proteins combined to decrease inflammatory factors in H:BM-M

Following the results of miRNA analysis, we analyzed gene ontology (GO) to understand the role and biological function of proteins related to these miRNAs. In a previous experiment, we confirmed that when EVs were pre-treated with HMEEC and then induced inflammation with LPS, the result of rapidly reducing the inflammatory factor after 1h by EVs derived from co-culture media (Fig.4). It was assumed that there would be information on useful molecules in H:BM-M, which significantly reduced the gene expression of TNF-, COX-2, IL-1, and IL-6 in inflammation-induced cells. Therefore, we analyzed the expression of miR-638, which was highly expressed in H:BM-M.

**Fig. 7:**
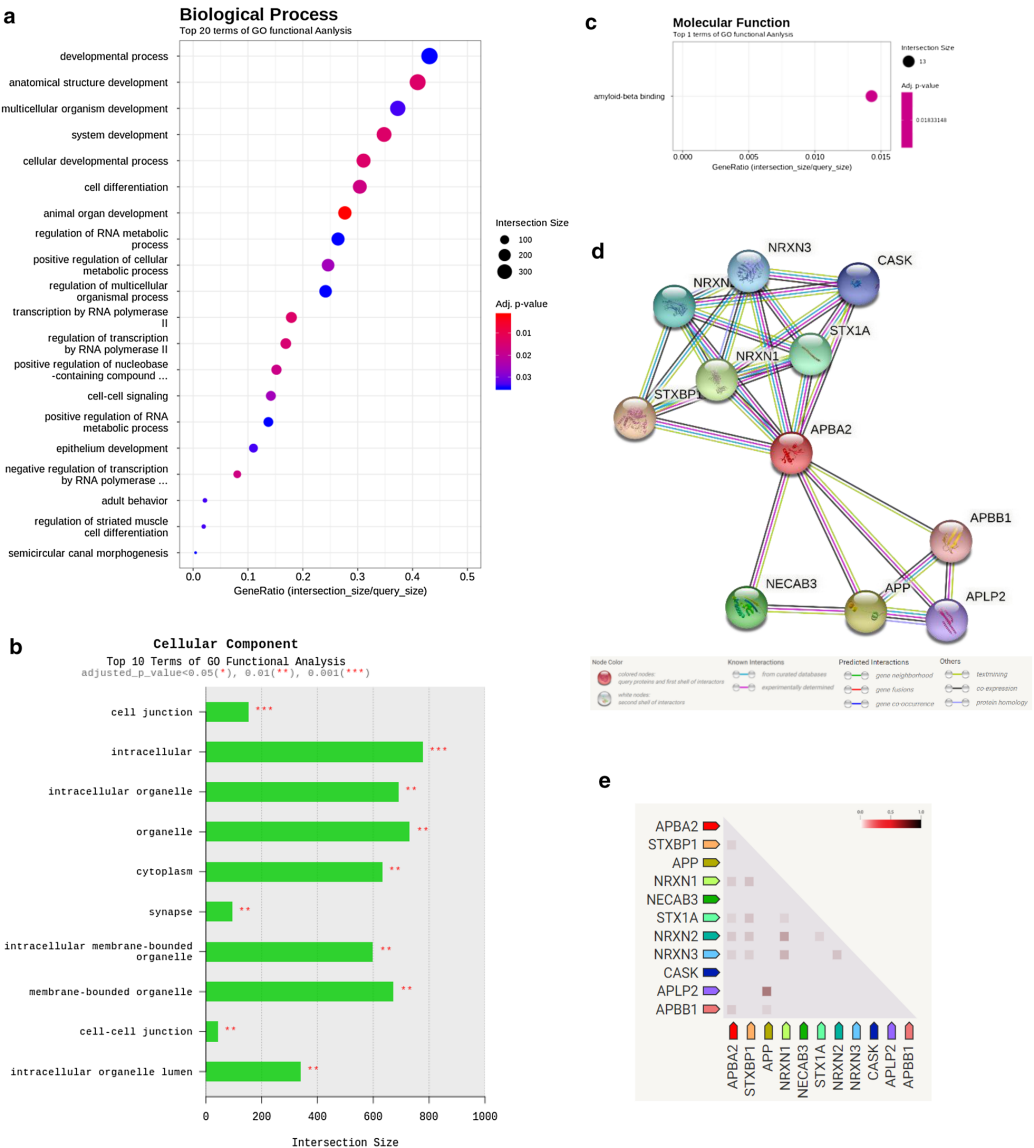
Gene ontology analysis by miRNA in EVs derived from co-culture media between BM-M and H:BM-M. **a** The analysis of biological process, **b** The analysis of cellular component, **c** The analysis of molecular function, **d** The analysis of protein connection in APBA2, **e** The comparison analysis of APBA2 with other proteins

First, most of the proteins expressed for miR-638 included factors that affected the development of intracellular proteins in donor cells. It is also involved in RNA transcription polymerase-related factors and cell-cell signaling. In addition, there was a small expression level related to the factors involved in differentiation (Fig.7a).

Next, the relevance of the cellular component was analyzed, and an association with a total of 10 organelles was shown. The predicted proteins were mainly related to the intracellular, membrane, and cytoplasm (Fig.7b). In addition, among them as a result of analyzing molecular function, it was confirmed that the factors related to protein binding significantly increased. In particular, the expression of the amyloid-beta binding protein was found to be the largest (Fig.7c). It has been reported that amyloid-beta A4 precursor protein-binding family A member 2 (APBA2) is involved in synaptic transport and junction of neurons [35], but they are also involved in improving immunity and cell regeneration [36]. We hypothesized that the anti-inflammatory effect of HMEECs was increased by the expression of miRNA-638 in H:BM-M and APBA2 could be expected to be a candidate connection protein (Fig.7d). APBA2 also showed an association of 0.989 with STXBP1 and a low association with NRXN1 and STX1A (Fig.7e). Therefore, we concluded that BM-MSCs significantly increased the expression of APBA2-related proteins in EVs in the medium by exchanging substances with HMEEC, thereby improving the anti-inflammatory effect of HMEECs.

## Discussion

Research on the development of therapeutic materials using human MSC-derived EVs has been actively reported in the field of regenerative medicine and anti-inflammatory materials [5, 37, 38]. Representative human stem cells of various origins, such as adipose tissue, bone marrow, umbilical cord, and tonsil, have been reported [5, 14]. Interestingly, some studies have reported that MSCs derived from different tissues have different payloads to communicate with recipient cells as donor cells by using EVs with a determined size of less than 100 nm [5, 14]. However, there has not been a study comparing the components of MSC-derived EVs of different origins to discover therapeutic materials for inflammatory diseases or cancer diseases.

We hypothesized that EVs derived from different origin influence different inflammatory effects on one recipient cell. Here, we would like to propose that two new facts. (1) The anti-inflammatory effects of EVs derived from MSCs of different origins are indicated by differences in payloads. (2) Crosstalk between cells by co-culture can improve anti-inflammatory effects by altering the payload of EVs. MSCs were extracted from three different origin (Adipose tissue, Bone marrow, and Tonsil), and the change in payload of EVs obtained by co-culture with middle ear epithelial cells (HMEEC) in transwells was analyzed. Adipose tissue (AD)-derived MSCs express mRNAs related to transcription factors, angiogenesis, and adipogenesis [18, 27]. Bone marrow (BM)-derived MSCs are widely known for their regenerative, immune stimulatory, and related factors, such as calcium signaling and cytoskeletal genes [20]. Tonsil (T)-derived MSCs have been reported as cells whose clinical therapeutic efficacy has been confirmed through studies in which the expression of inflammatory cytokines was increased through injection in acute and chronic colitis models [28, 39]. In this study, using a model that causes otitis media (OM), a disease with a high incidence in children, we confirmed the efficacy of MSC-derived EV to reduce inflammatory factors (TNF-, COX-2, IL-1, and IL-6). The expression of inflammatory factors, induces immune regulation, mucosal changes, and inflammatory responses, leading to the development of OM [26]. Among these, *TNF-*, which plays an important role in the induction of an inflammatory cascade, is known to induce OM [39]. *COX-2* is the major enzyme that converts arachidonic acid to prostaglandin H2 to produce inflammatory mediators. The expression level of *COX-2* was upregulated in the middle ear of patients with OM compared to that in healthy subjects [40]. It has been reported that it can damage hearing if the inflammation of HMEECs increases significantly [41]. OM is mainly focused on in clinical research, but this finding of molecules in stem cell-derived EVs can be valuable in development of a new material by reducing the death of middle ear cells and ROS reduction by inflammatory factors [41].

The viability of HMEECs was over 98% during co-culture with AD-MSCs, BM-MSCs, and T-MSCs, as verified by FACS (Fig.1b), confirming that no toxic substances were generated. The expression levels of inflammatory markers significantly increased after 24h of exposure to 1g/mL LPS, and the cell viability also decreased to less than 80%. We found that apoptosis was induced by exposure to LPS after 24h (Fig.2).

To analyze the change in payload in the MSC-derived EVs that we hypothesized, donor MSCs and recipient HMEECs were co-cultured in trans wells. The EVs were separated from the co-culture medium by centrifugation, and it was confirmed that ROS production induced by LPS was decreased by EVs in HMEECs (Fig.3d). Based on these results, it was proved that the MSC-derived EVs, which are labeled AD-M, BM-M, and T-M in a single culture, contained factors capable of reducing ROS levels. Furthermore, there might be several useful factors expressed in co-culture media because large amounts of CD9, CD63, and CD81 (EV markers) were found after co-culture of 24h (Fig.3e and f). In other words, the co-culture conditions showed that EV generation can be increased by the crosstalk between donor and recipient cells, suggesting that the payload can be changed.

It was confirmed that the size of EVs in the single medium and co-culture media was less than 100 nm (Fig.3h and i), and the levels of inflammatory factors expressed by LPS were decreased by EVs. The EVs derived from MSC single culture media (AD-M, BM-M, and T-M) reduced the mRNA expression levels of *TNF-, COX-2, IL-1*, and *IL6* by half (Fig.4). Interestingly, among the EVs derived from co-culture media (H:AD-M, H:BM-M, and H:T-M), H:BM-M showed the greatest efficiency in reducing inflammatory factor expression until zero by pretreatment with HMEECs for 3h. Therefore, we hypothesized that some molecules had been delivered to the recipient cells, which is related to reduced expression of inflammatory factors.

**Fig. 8:**
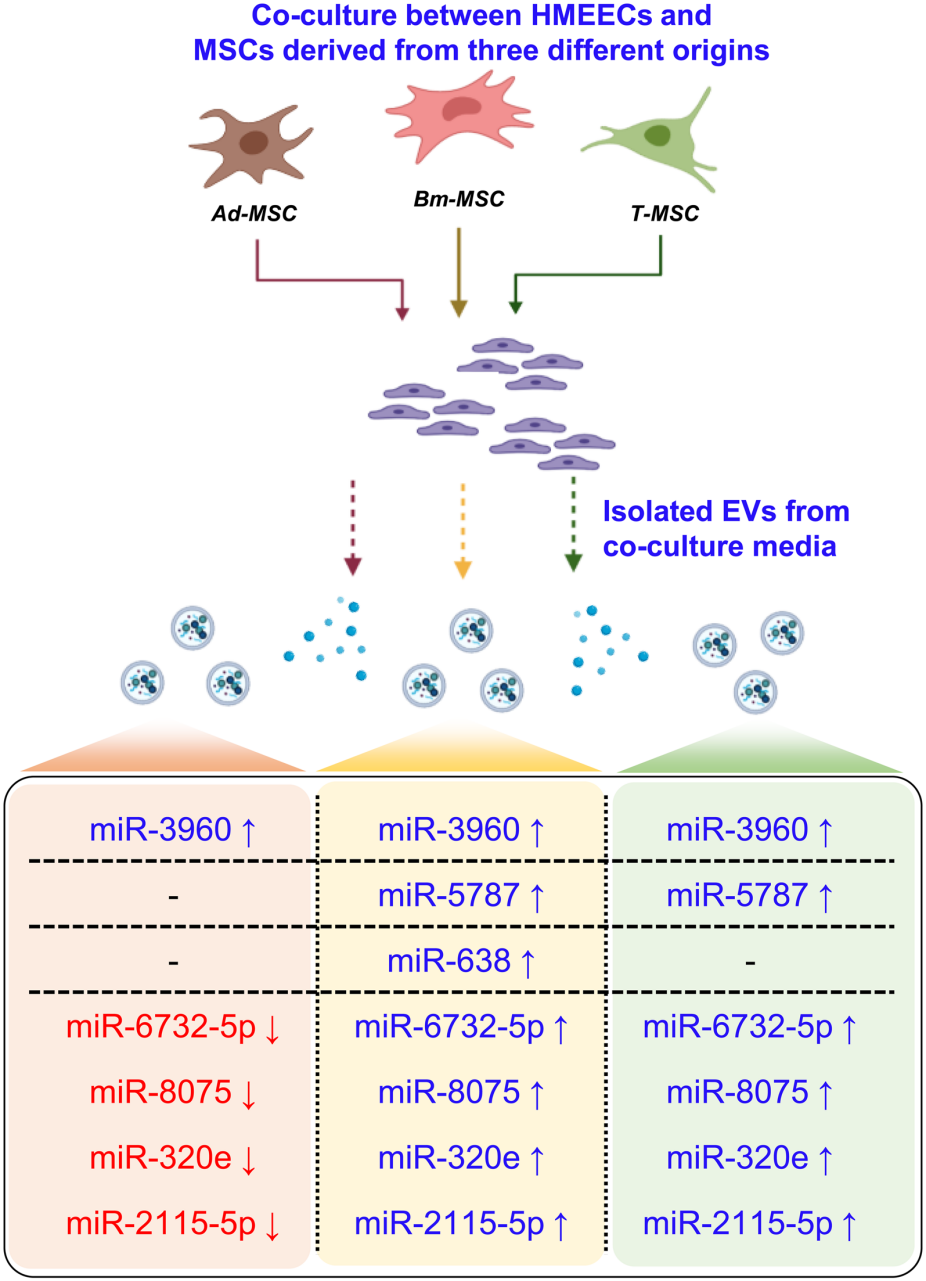
A schematic diagram of miRNAs in EVs derived from the co-culture media

The useful factors contained in EVs and some miRNA databases were analyzed by miRNA analysis (Fig.5). Using miRNA analysis, we found that 161 miRNAs were expressed under the three co-culture conditions, and these were related to the effects of immunity and proliferation on the cells (described in Table1). miR-3960 was highly expressed in H:AD-M, H:BM-M, and H:T-M. The predicted mRNA levels were analyzed for POU class 3 homeobox 3 (*POU3F3*) [42], protocadherin alpha 2 (*Pcdha2*) [43], ceramide synthase 1 (*CERS1*) [44], early growth response (*EGR*) [45], and macrophage migration inhibitory factor (*MIF*) [46]. miR-5787 was highly expressed in both H:BM-M and H:T-M cells. The predicted mRNA was analyzed for phosphofurin acidic cluster sorting protein 1 (*PACS1*) [47], protein phosphatase 1 regulatory subunit 7 (*PPP1R7*) [48], visual system homeobox 2 (*VSX2*) [49], SMAD family member 2, SMAD family member 3 [50], and synaptotagmin 1 (*SYT1*) [51]. In addition, miR-6732-5p, miR-8075, miR-320e, and miR-2115-5p expression decreased in the H:AD-M group and increased in the H:BM-M and H:T-M groups (Fig.8). Interestingly, miR-638, which significantly reduced *TNF-, COX-2, IL-1*, and *IL-6* (Fig.4) expression, showed increased expression only in H:BM-M. One of the predicted mRNAs analyzed Solute carrier family 25-member 23 (*SLC25A23*) and Adhesion G protein-coupled receptor E5 (*ADGRE5*) was reported to be involved in both adhesion and signaling processes [45]. *SLC25A23* reportedly decreases mitochondrial Ca (2+) uptake and reduces cytosolic Ca (2+) clearance after histamine stimulation [51]. In addition, approximately 100 predicted mRNAs were analyzed, and we tried to select the most related proteins through gene ontology analysis. We hypothesized that miR-638-related protein inhibited the increase in inflammatory factors in HMEECs; therefore, we predicted that it was *APBA2* (Fig.7c). It is cautious to predict proteins that have organic relationships in cells, but we found that *APBA2* can play a role in binding and transmitting factors related to binding to membranes containing immunity and differentiation [36]. *APBA2* encodes a cell surface receptor and transmembrane precursor protein that is cleaved by secretases to form a number of peptides. In addition, two of these peptides are antimicrobial peptides, which have been shown to have bactericidal and antifungal activities. Mutations in this gene are associated with autosomal dominant Alzheimers disease and cerebral arterial amyloidosis (cerebral amyloid angiopathy). Several transcriptome variants have been discovered that encode several different isotypes of this gene [52]. As reported for EVs to communicate between cells, a major difference among vesicle subtypes is their origin, while microvesicles and ectosomes bud from the plasma membrane, EV formation begins on early endosomes [6, 7]. After maturation in multivesicular bodies through the invagination of the endosomal membrane and formation of intraluminal vesicles (ILVs), EVs are released by the fusion of ILVs with the plasma membrane [14]. The specific proteins incorporated into ILVs are regulated mainly by the endosomal-sorting complex required for transport (ESCRT), of which there are four: ESCRT-0, I, II, and III. As a result, we discussed that binding proteins, such as *APBA2*, can induce and stimulate the transport of inflammatory factors by connecting the cellular membrane. In a study that reported the activity of *APBA2*, it was reported in patients with age-related macular degeneration (AMD), and autophagy factors were identified by treating ARPE-19 cells with soluble amyloid with an oligomer [53]. Autophagy activated in ARPE-19 cells treated with *APBA2* altered the expression pattern of LC3 by creating a phagocytic compartment and activating p62, thus clarifying the potential ambiguity mechanism of retinal cells. It has been reported that *APBA2* is involved in the MAPK signaling pathway and it alters cell viability, migration, and invasion [52]. However, the relationship between amyloid activity and neuroinflammation has been reported [54]; the role of inflammation in auditory hearing loss relief should be explored in further studies.

**Table 1:** List of predicted target mRNAs by analysis of miRNAs in EVs derived from the co-culture media (AD-H:AD, BM-H:BM, TM-H:TM)

miRNA	Predicted target mRNA		Function and detail	Reference	
	Symbol	Full name		ExoCerta, Vesiclepidia	PMID
(a) Predicted target genes on the increased expression of miRNA under three conditions (AD-H:AD, BM-H:BM, TM-H:TM)					
miR-3960	POU3F3	POU class 3 homeobox 3	Regulation of oncogenic signaling pathway Interaction with SMAD protein and the tumor suppressor PTEN	VP_5455	[42]
	Pcdha2	Protocadherin alpha 2	Establishment and function of specific cell-cell connections. Demonstrate an organization similar to that of B-cell and T-cell receptor gene	ExoCarta_393086 VP_393086	[43]
	CERS1	Ceramide synthase 1	Encodes a ceramide synthase enzyme, which catalyzes the synthesis of ceramide Encodes growth differentiation factor from a monocistronic mRNA	ExoCarta_10715 VP_10715	[44]
	EGR	Early growth response	May also play a role in a wide variety of processes including muscle development, lymphocyte development, endothelial cell growth and migration, and neuronal development	ExoCarta_13654 VP_1959	[45]
	MIF	Macrophage migration inhibitory factor	Encodes a lymphokine involved in immunoregulation, cell-mediated immunity, and inflammation	ExoCarta_4282 VP_4282	[46]
(b) Predicted target genes on the increased expression of miRNA under two conditions (BM-H:BM, TM-H:TM)					
miR-5787	PACS1	Phosphofurin acidic cluster sorting protein 1	Plays a role in Nef-mediated downregulation of cell surface MHC-I molecules to the TGN, thereby enabling harmful factors to escape immune surveillance	ExoCarta_107975 VP_107975	[47]
	PPP1R7	Protein phosphatase 1 regulatory subunit 7	Encodes a protein subunit that regulates the activity of the serine/threonine phosphatase, protein phosphatase-1	ExoCarta_5510 VP_5510	[48]
	VSX2	Visual system homeobox 2	Encodes a homeobox protein originally described as a retina-specific transcription factor	ExoCarta_338917 VP_338917	[49]
	SMAD2, SMAD3	SMAD family member 2, SMAD family member 3	Functions in the transforming growth factor-beta signaling pathway Cell proliferation, tumor suppressor	ExoCarta_4088 VP_4088	[50]
	SYT1	Synaptotagmin I	Ca(2+) sensors in the process of vesicular trafficking and exocytosis	ExoCarta_6857 VP_6857	[51]
(c) Predicted target genes on the increased expression of miRNA on BM-H:BM					
miR-638	ADGRE5	Adhesion G protein-coupled receptor E5	Involved in both adhesion and signaling processes early after leukocyte activation	ExoCarta_976	[46]
	SLC25A23	Solute carrier family 25-member 23	Decreases mitochondrial Ca (2+) uptake and reduces cytosolic Ca (2+) clearance after histamine stimulation	ExoCarta_79085 VP_79085	[51]
	Cela3b	Chymotrypsin-like elastase family, member 3B	Functions in the intestinal transport and metabolism of cholesterol Little elastolytic activity	ExoCarta_67868 VP_67868	[47]
	KDSR	3-ketodihydrosphingosine reductase	Catalyzes the reduction of 3-ketodihydrosphingosine to dihydrosphingosine	ExoCarta_2531 VP_2531	[55]
	EMILIN3	Elastin microfibril interfacer 3	Developing gonads and osteogenic mesenchyme	ExoCarta_90187 VP_90187	[56]
(d) Predicted target genes for miRNa with increased expression on BM-H:BM,TM-H:TM and miRNA with decreased on AD-H:ADM					
miR-320e	LAPTM4A	Lysosomal protein transmembrane 4 alpha	Transport of small molecules across endosomal and lysosomal membranes.	ExoCarta_9741 VP_9741	[57]
	PNRC1	Proline-rich nuclear receptor coactivator 1	RNA polymerase (pol) II-transcribed genes by functioning as a nuclear receptor coactivator	ExoCarta_10957 VP_10957	[50]
	SKA3	Spindle and kinetochore associated complex subunit 3	Component of the spindle and kinetochore-associated protein complex that regulates microtubule attachment to the kinetochores during mitosis	VP_221150	[46]
	TMEM47	Transmembrane protein 47	Localized to the ER and the plasma membrane	ExoCarta_83604 VP_83604	[58]
	SEMA6D	Sema domain, transmembrane domain (TM), and cytoplasmic domain, (semaphorin) 6D	Secreted and membrane associated proteins Axon pathfinding, fasciculation and branching, and target selection	VP_80031	[59]
miR-8075	TMOD2	Tropomodulin 2	Actin-capping protein for the slow-growing end of filamentous actin	ExoCarta_29767 VP_29767	[22]
	ADCY1	Adenylate cyclase 1	Adenylate cyclase gene family that is primarily expressed in the brain Ca(2+)/calmodulin concentration	ExoCarta_107 VP_107	[48]
	CD1B	CD1b molecule	Primarily lipid and glycolipid antigens of self or microbial origin to T cells	ExoCarta_910 VP_910	[60, 61]
	OLFM1	Olfactomedin 1	Neurogenesis, neural crest formation, dorsal ventral patterning, cell-cell adhesion Cell cycle regulation, tumorigenesis, and signaling pathways (Wnt, BMP)	VP_10439	[62, 63]
	PRTG	Protogenin	Development of various tissues, especially neurogenesis	VP_283659	[46]
miR-6732-5p	OAS2	2-5-Oligoadenylate synthetase 2, 69/71kDa	Innate immune response to viral infection	ExoCarta_4939 VP_4939	[61]
	STX17	Syntaxin-17	Control of autophagosome membrane fusion with the lysosome membrane		[63]
	KLK3	Kallikrein related peptidase 3	GPCR signaling pathways, translation Non-genomic (rapid) action of androgen receptor.	VP_354	[64]
	DYNC1LI1	Dynein, cytoplasmic 1, light intermediate chain 1	Intracellular vesicle trafficking	ExoCarta_51143	[65]
	ABCC5	ATP-binding cassette, sub-family C (CFTR/MRP), member 5	Resistance to thiopurine anticancer drugs, 6-mercaptopurine and thioguanine functions	ExoCarta_10057 VP_10057	[66]
miR-2115-5p	ATL3	Atlastin GTPase 3	Network of interconnected tubules of the endoplasmic reticulum	ExoCarta_25923 VP_25923	[67]
	USP49	Ubiquitin specific peptidase 49	Epigenetic transcriptional activation and acts as a regulator of mRNA splicing.	ExoCarta_25862 VP-25,862	[46]
	TTPAL	Alpha tocopherol transfer protein like	Stimulates the movement of vitamin E between membrane vesicles in vitro and facilitates the secretion of tocopherol from hepatocytes	N/D (Not detected)	[68]
	CERS6	Ceramide synthase 6	Cell proliferation, differentiation, apoptosis, and senescence	ExoCarta_253782 VP_253782	[69]
	CD247	CD247 molecule	Encodes an immune inhibitory receptor ligand that is expressed by hematopoietic and non-hematopoietic cells, such as T cells and B cells, and various types of tumor cells	ExoCarta_919 VP_919	[70]

MSCs were extracted from three tissues and the changes in miRNA payload in EVs and the anti-inflammatory effect of MSC-derived EVs were analyzed in this study. In particular, to improve the anti-inflammatory effect of epithelial cells that induce inflammation, EVs of epithelial cells and MSC co-culture medium were extracted, and the payload was confirmed to change. This study was conducted based on the essential meaning of EV as an intercellular messenger to confirm the payload of HMEEC-derived EV and MSC-derived EV. We believed that the EVs derived from co-cultured media generated containing new molecules by the cell-to-cell communication process, so EVs in a single culture medium was used as controls. The payload in the EVs showed significantly different when co-culture between HMEEC and MSCs in Fig.5. However, it was not possible to prove the factors expressed from different cell line in the single culture medium, we tried to explain reflecting the biological characteristics of EV for future studies. We discussed that the mechanism of cross-talk between different cell lines makes it possible to express unique biomarkers. Our study predicts that the change in the payload in EVs expressed by co-culture with stem cells can be used in various disease models. Therefore, the co-culture of stem cells into donor and recipient cells lead to the expression of new factors, which could prove valuable in the field of regenerative medicine and material development.

## Data Availability

Not applicable.
